# Supplementary material for: Learning curve and surgical outcome of robotic assisted colorectal surgery with ERAS program
Source: Sci Rep. 2022 Nov 29;12:20566. doi: 10.1038/s41598-022-24665-w (PMC9709162; doi:10.1038/s41598-022-24665-w)
Supplement: Supplementary file 1 — Supplementary Information 1. [file 41598_2022_24665_MOESM1_ESM.zip › LSC Raw data-Ñ[▒K/070/N_ñw╝╨░O▒Kñσ.pdf]

W83 / W83

臺中榮民總醫院
手術室護理記錄

[redacted]

基本資料

- 室別： 開刀房-06室，手術科別： CRS，手術類別： 常規，日期： 2020/02/20
- 進入時間： 1145 ； 麻醉開始時間： 1150 ； Time Out時間： 1240 ； 劃刀時間： 1241 ； 手術結束時間： 1455 送出時間： 1515 ；
- 麻醉方式： GE
  診斷： Malignant tumor of rectum FOR LAP CAA
  手術名稱： 1.Low anterior resection with colo-anal anastomosis 2.Laparoscopy
  術前預防性抗生素：
  - 抗生素名稱　： Veterin 1GM IVD/由麻醉護理執行
  - 時間： 1230
  - 上次簽章: 彭馨慧

  ，追加預防性抗生素：
- 手術醫師： 蔣鋒帆，江世偉，邱俊凱，陳思如
- 麻醉醫師： VS 徐煥
-
  - 刷手護士A ： 吳雅華，時間： 1145-1500，刷手護士B ： 戴宛榛，時間： 1145-1500，接替刷手一： 朱詠暄，時間： 1500-1515
  - 流動護士A ： 彭馨慧，時間： 1145-1300，接替流動一： 吳若綺，時間： 1300-1500，接替流動二： 洪紹瓊，時間： 1500-1515

皮膚狀況

A-V Shunt： 無
術前： 完整
　記錄者： 上次簽章: 彭馨慧
術後： 完整
　記錄者： 上次簽章: 吳若綺

手術準備

手術臥式 單一手術臥式

-
  - 手術臥式： 截石臥式
  - 使用附件： 溫毯 38 ℃，頭圈
  - 皮膚準備： 10%Aq Betadine，Hibiscrub，其它： 2%chlorhexcidine
  - 電燒： 使用電燒: 電極片位置： 其它： 使用床上電極墊，記錄者： 上次簽章: 彭馨慧

止血帶T： 無

植入物

- 植入物名稱/號碼/數量/部位： Ethicon Endo clip/20釘/colon surgiwrap/100mm*130mm/骨盆腔

，記錄者： 上次簽章: 吳若綺

標本送檢

- 細菌培養 無
- 細胞學 無
- 冰凍切片 無
- 病理標本
  組織名稱一： rectum
  ，
  組織名稱二： IMA root
  ，
  組織名稱三： distal cutend
- 截肢 無

標本交付 標本： ? 袋，標本： 零 盒，標本： 零 瓶，細胞學： 零 管，Culture Tube： 零 管，被交付醫師： R 邱俊凱，

- 記錄者： 上次簽章: 吳若綺
- 時間： 1500

傷口術後敷料

傷口敷料： 傷口敷料： 無

- 請於圖上標示部位：

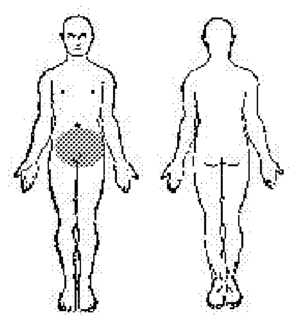

腹部腹腔鏡肆傷口

- 傷口敷料： menpor覆蓋

導管

導管	天數	導管資訊	插入日期	插入時間	更換日期	拔除日期	拔除時間	拔除原因	預計拔除/更換	預計拔除/更換日期
Foley(1)	1	號碼:16 Fr.; 部位:尿道;	2020/02/20	1230						
Penrose Drain(1)	1	號碼:12 mm.; 部位:骨盆腔;	2020/02/20	1430						
Sump NG Tube(1)	1	號碼:16 Fr.; 部位:胃;	2020/02/20	1235						

用物計數

- 手術中出血量： minimal c.c.
- 手術中尿量： 150 c.c.

用物計數： 多切口同時縫合

- 1.器械： 正確，2.縫針： 正確，3.刀片： 正確，4.紗布： 正確，5.腹部墊： 未使用，6.紗球： 未使用，7.PEANUTS ： 未使用，8.紗條： 未使用，9.絲帶： 未使用，10.針頭： 未使用，11.Vessel loop： 未使用，12.Boots： 未使用，13.Jelco： 未使用，14.棉條： 未使用，
    - 第一次：
        - 刷手護士 ： 吳雅華
        - 流動護士： 上次簽章: 吳若綺
        - 時間： 1410

      ,
    - 第二次：
        - 刷手護士 ： 吳雅華
        - 流動護士： 上次簽章: 吳若綺
        - 時間： 1425

      ,
    - 第三次：
        - 刷手護士 ： 吳雅華
        - 流動護士： 上次簽章: 吳若綺
        - 時間： 1435

備註

備註： NIL

- 記錄者： <u>洪紹瓊</u>
- 時間： 1515

填寫護理人員 洪紹瓊

正式摘要

W83 / W83

doctorRank:B
